# Open Letter—Answer to Dr. Paquin

**Published:** 1891-01

**Authors:** D. E. Salmon

**Affiliations:** Washington


### OPEN LETTER—ANSWER TO DR. PAQUIN.

EDITOR JOURNAL OF COMPARATIVE MEDICINE AND VETERINARY ARCHIVES:—

DEAR SIR:—The open letter contributed by Dr. Paquin to the December number of the JOURNAL is remarkable in several respects, but calls for attention chiefly because of the misrepresentations which it contains. It is somewhat remarkable, considering the interest manifested, that the Doctor hesitated to enter into a full discussion at the Chicago meeting of the U. S. Veterinary Medical Association, especially as his investigations were criticised in the report of one of the committees, and every one conceded him the right of making the fullest possible reply. It was generally supposed that he defended his position as well as the merits of the case would permit, and the reader of his letter will search in vain for any fresh arguments in his favor. Instead of bringing additional evidence to support his investigations, his object now appears to be rather to assume the rôle of injured innocence, by posing as a modest investigator who has been unjustly criticised and wronged without excuse.

Now what are the facts? In the brief remarks on Texas fever, contained in the paper which I read at Chicago, Dr. Paquin's name was not mentioned, nor were his investigations discussed. With the report made by the Chairman of the Committee on Diseases I had nothing to do, neither having been consulted in its preparation nor having any idea of its contents until it was read. Whether that report was too harsh in its references to Dr. Paquin can be decided by each individual reader for himself. Dr. Paquin was there to present his own side of his case and was listened to respectfully. And when the investigations of the Bureau of Animal Industry were commented on by both of the principals in the discussion, I claimed the same right to defend my work that was freely granted to him. It was a free and open discussion, where we all endeavored to give the reasons for our views, and Dr. Paquin has no more reason to "protest against the positiveness and exclusiveness" with which Dr. Clement and myself explained our views, than we have to enter the same protest against his remarks. In either case a protest is ridiculous, for the most important business of a scientific meeting is to bring out discussions and enable investigators to present their evidence and views with all the clearness and positiveness at their command, due allowance being made for that fairness and courtesy which should always characterize scientific gatherings.

Dr. Paquin also protests against "the uncalled-for allusion to jealousy which was hurled from such a high official position as Dr. Salmon's." This is making a very large mountain out of a small mole-hill. The report of the discussion as taken by the stenographer and printed in the JOURNAL for November bears me out in the assertion that no "allusion to jealousy" was hurled at our super-sensitive friend. The only time I used the word was in the following paragraph, called out by the somewhat personal character of the discussion which had taken place between Drs. Paquin and Clement, and the references made to my own work. I trust your readers will examine the language carefully and see for themselves just how much cause the gentleman has for printing an open letter of protest. The remarks were as follows:

"I regard it as unfortunate that there should be so much feeling between different investigators in the same field of work. There is room enough for all of us to work and surely enough to be done, and the important questions connected with these diseases will be solved none too soon if we all work and strive together to throw what light we can upon the subject. As far as I am concerned, I feel no jealousy in regard to the work of Dr. Paquin, or any other investigator, and I should feel great regret if I knew there was any such jealousy in regard to my work, because I appreciate the importance of all working together. We will naturally get some different results, but when we come to put these together and study them, and draw our conclusions, we will probably see that each from his standpoint, from his methods of work, is able to throw light upon the problems which we all desire so much to understand."

After these remarks were concluded Dr. Paquin arose and expressed his pleasure at the spirit of the discussion, saying it was exactly what he wanted!

The Doctor goes on to say, in his letter, "Dr. Salmon tells us that *tics* carry Texas fever to the North. We had explained that in our bulletin, but Dr. Clement and he ignored it, as they did every other new point of practical value found in our bulletin." If Dr. Paquin had reflected a moment he would have remembered that I made no attempt to review his bulletin. My remarks were called out by the discussion and were confined to the assertions therein made. If he wished his investigations in regard to *tics* discussed he had every opportunity to bring up the subject, but having failed to do so, he has no right to blame others for his delinquencies.

Now that he insists that he has explained the action of ticks in his bulletin, we will briefly review his utterances on that point and show how much (or how little) his investigations in that direction amounted to. In the conclusions on page 55 (Texas Fever

Bulletin) he says: "Ticks and the feet of cattle are capable of carrying the germs to distant lands." What does this mean? Does he intend to give the impression that ticks when not carried by cattle may migrate to distant lands and infect them? Or that they only infect lands when carried by Southern cattle? In either case, how does he know that ticks are capable of carrying the germs to distant lands? Does he give an account of any observations or experiments which demonstrate this assertion? Not at all.

By retracing our way through his bulletin we find on page 52 this statement: "That ticks full of blood from infectious Southern cattle, may, it seems, scatter the germs on our lands, though ticks *of themselves cannot* convey the disease." Here he is entirely wrong. Not only can ticks convey the disease themselves and directly, but ticks which are not full of blood can convey it. Our experiments prove that ticks which have never been on infectious Southern cattle can produce Texas fever, for we have produced it with young ticks which were hatched in the laboratory from the eggs of ticks gathered from Southern cattle.

How then does Dr. Paquin get the idea that ticks may carry the germs at all? Turning to page 45 of his bulletin we find the remainder of the story, and with this we have, I believe, his complete writings in explanation of the relation of ticks to the etiology of Texas Fever. He says: "That is not all; we have found the parasites also in *ticks bloated with blood of infectious Southern cattle.* So this must be added to the list of sources." Here we have the full explanation. In examining the contents of ticks bloated with the blood of Southern cattle he saw what he believed to be the germ of Texas fever. But how did he know that ticks would scatter this germ in a virulent condition on pastures? How does he know that what he saw was really the germ of Texas fever? No intelligent bacteriologist in these days pretends to identify such a micro-organism by its form alone, no matter how constant and unchangeable this may be. But Dr. Paquin asks us to believe in his ability to identify this germ under such unfavorable conditions as when it is mixed with the debris of partially digested blood corpuscles and the various micro-organisms which may normally be present in the digestive organs of the tick. This would be drawing heavily on our credulity under any circumstances, but when we look at his drawings and see that his germ is represented as a single coccus and a double coccus, a short thin rod and a short broad rod, a long thin rod and a long broad rod, in fact as having about all the forms which ordinary putrefactive bacteria assume, we cannot possibly imagine how he could dis-inguish by microscopic examination between the germ which he thinks produces Texas fever and any of the ordinary bacterial species which are to be found all around us.

Now, it is the repeated instances of this kind in which Dr. Paquin assumes his ability to do what no modern bacteriologist would pretend to be possible under the circumstances, which has led some of his confreres to criticise his work and others to ignore it. In the discussion at the Chicago meeting, without for the moment saying who was right or who wrong, I asserted very positively that the germ described and figured by Dr. Paquin could not be the same as that we were working with in the Bureau of Animal Industry. The germs are radically different, and no competent person can look at the protozoon in the blood globules of Texas fever cases, and then read Paquin's description and consult his figures, and still believe that the Doctor had ever seen our germ at the time his bulletin was written. No matter whether he classified his germ or not, no matter to what kingdom of nature he assigned it, his text and his figures demonstrate conclusively that he was working with an entirely different kind of organism. Now, if it turns out that the disease is caused by the rod-shaped organisms which he described, and which can be cultivated on blood serum, on potato, in beef broth, etc., and which may be found in a motile condition in the blood, bile and other liquids, as he describes, then we will not contest with him the honor of having discovered the cause of the disease. But if, on the contrary, the germ is not rod-shaped, if it has no motility except the amœboid movements of the protozoa, if it cannot be cultivated according to the ordinary bacteriological methods, if it cannot be distinguished and identified by present methods of research outside of the red blood globules, then it is absurd for Dr. Paquin to assert that it is the same organism with which he worked and which he tried to describe in his bulletin. There are other points in the bulletin which have been, as Dr. Paquin says, ignored. These are notably his alleged identification of the germ in the surface soils, pond waters and grasses of the Southern States; his statement that germs just excreted from the animal body are not dangerous, but must have a resting period before they regain their virulence; his identification of the germ in the organs of insusceptible Southern cattle from the infectious districts and in the organs of their calves before and after birth; his cultivation of these germs and the possibility of successfully using the cultures for preventive inoculation. In the first place, it is physically impossible for any man to have carefully investigated and decided all the questions, which he in his bulletin claims to have decided, in the few months over which his work extended. In the second place, several of his conclusions are extremely improbable when considered in the light of the present knowledge of germs and germ diseases, and are at variance with the results which we have positively established and which can be easily repeated by any one. In the third place, he admits that his bulletin was written for the general public and not for scientists, and that he purposely withheld the details of his methods of making and using his vaccine virus to prevent others for the present from repeating his experiments in this direction. Under such circumstances what right has he to complain if his work is ignored, until these questions are settled by scientists who are willing to publish their experiments with sufficient details to allow other investigators to repeat them and test the accuracy of the conclusions?

*Washington, Dec. 19th, 1890.* D. E. SALMON.

